# Protecting Clinical Trial Participants and Protecting Data Integrity: Are We Meeting the Challenges?

**DOI:** 10.1371/journal.pmed.1001234

**Published:** 2012-06-12

**Authors:** Susan S. Ellenberg

**Affiliations:** Perelman School of Medicine at the University of Pennsylvania, Philadelphia, Pennsylvania, United States of America

## Abstract

Susan Ellenberg discusses alternative approaches towards evaluating data as it accumulates in clinical trials, and to protecting the integrity and preventing undue risks to participants, as the trial continues.

**Summary Points**

- Although there is substantial consensus regarding the need for interim monitoring of certain types of trials, there is controversy about specific aspects of data monitoring.
- Approaches to ensuring independence of those who perform the interim monitoring and confidentiality of interim data vary substantially by type of trial and trial funder.
- The “independent statistician” model, involving a separate statistician to analyze interim data and report to the data monitoring committee (DMC), remains controversial but provides important protections of data integrity.
- Early stopping guidelines should be clearly understood and accepted by all parties, and only deviated from if there are unexpected findings that confound the overall benefit-risk assessment at interim analysis.
- Liability of DMC members is an important concern that has not been dealt with adequately by either commercial or government trial sponsors.

*This is one article in an occasional* PLoS Medicine *series on research integrity that examines issues affecting the ethics of health research worldwide.*

## Introduction

A long-standing tenet of clinical trial conduct is that the accumulating data must be monitored, if not continuously then at regular intervals during the trial. Such regular review helps to ensure that risks to participants are not greater than anticipated and that the study is being conducted appropriately. Early randomized trials were typically monitored by the investigator(s), the research funder (a government agency or a pharmaceutical company), or by a steering committee appointed by the funder [1], with no well-accepted quantitative criteria for decision-making and often with the accumulating data widely known.

The practices of data monitoring committees (DMCs; also known as Data and Safety Monitoring Boards [DSMBs]) have evolved substantially over the last 40 years. Statistical methods to guide early termination decisions have been developed [2]–[5]; the principle of confidentiality of interim data has become widely accepted; and the actual conduct of DMC meetings has become more standardized. Many publications, an international US National Institutes of Health (NIH) symposium [6], and at least three books [7]–[9] have addressed the philosophical and operational issues faced by DMCs; in the United States, the NIH and the US Food and Drug Administration (FDA) have established policies regarding DMC establishment and operation [10],[11], as have international organizations [12],[13]. A large study of DMC practices commissioned by the United Kingdom's Health Technology Assessment group has provided substantial detail regarding current DMC practices [14]–[16].

Despite the increased attention to DMC function, some issues remain controversial. Two of particular importance are 1) the extent to which DMC members and the statistician analyzing interim data and reporting to the DMC should be independent of the trial sponsor and investigators; and 2) the criteria that should be used to guide early termination decisions. A third issue, of increasing concern to scientists serving on DMCs, is that of liability of DMC members.

## Independence of the DMC and the Reporting Statistician

When selecting scientists to evaluate research findings, there is inevitable tension in seeking the most knowledgeable experts while minimizing real or perceivable conflicts of interest. The more knowledgeable and experienced a scientist is in a given area of research, the greater the likelihood that s/he will have some connection with any study in that area that could be perceived as a conflict of interest. (Similar tensions apply to selection of members of scientific and regulatory advisory committees, practice guideline committees, and reviewers for medical journals.) Currently, study investigators and employees of the manufacturer, who have clear conflicts of interest, are typically excluded not only from serving on the study DMC but also from attending sessions at which interim results by treatment group are presented and discussed. Other conflicts are more subtle. Should someone holding any amount of stock in a company be permitted to serve on a DMC for a study evaluating that company's products? What about someone serving as investigator in another study of the product, or any product, from the sponsoring company? Or an investigator serving on the company's speaker's bureau? Such individuals are often, but not always, excluded from serving on a DMC because such individuals might favor, or be perceived to favor, an outcome aligned with their personal interests. (When the clinical trials of rofecoxib [Vioxx], a COX-2 inhibitor that was ultimately withdrawn from the market due to adverse cardiovascular effects, were closely examined, it was discovered that the chair of the DMC of one important trial had nontrivial financial relationships with the product's manufacturer [17]. This revelation surely added to the belief of many, fairly or unfairly, that the chair, perhaps subconsciously, might not have evaluated the safety data as rigorously as he otherwise would have.)

Of course, the fact that an investigator holds stock in a company, or that s/he serves as a scientific advisor to the company for other purposes, does not necessarily imply that that investigator will be influenced by that connection to give more weight to protecting the company than study participants, but this can never be proven one way or the other. Concerns about unwanted influence on DMC members have increased to the point that some have advocated removing the selection of DMC members from the hands of companies entirely, placing that authority in an independent public body [18]. This solution may be overly extreme—it is difficult to imagine a single organization being able to effectively constitute appropriately expert DMCs for all trials run by the pharmaceutical industry—but more attention is needed to avoid conflicts of interest. Two possible approaches would be to intensify institutional review board (IRB) or FDA oversight of member selection. It seems unrealistic to expect multiple IRBs to review a trial's DMC membership in sufficient detail to identify conflicts of interest, but if central IRBs became standard in multisite trials, IRB review of the DMC membership, including financial disclosures, with the authority to reject members who appeared too close to the trial sponsor or had other significant conflicts, could be one solution. Alternatively, the FDA could review a company's proposed DMC membership, in the same way it reviews potential members of FDA advisory committees, and disapprove any member with an apparent conflict of interest. Implementation of either of these options would be challenging. Currently, while many have advocated central IRBs for multisite trials we are far from consensus as to whether this would be desirable [19],[20]. It could be more straightforward for the FDA to take on this oversight, but it would add substantially to the resources required for regulatory review.

DMCs for trials sponsored by noncommercial funders do not necessarily operate in the same way as DMCs for industry trials. For example, the widely accepted prohibition of sponsor representatives participating in DMC meetings with access to the unblinded interim data has not been applied by most US government sponsors. Wittes et al. [21], in describing the monitoring of the Women's Health Initiative (WHI) clinical trials, noted the unwillingness of the US National Heart and Blood Institute (NHLBI), whose representatives had access to the interim data and participated fully in the monitoring sessions, to permit the committee to discuss recommendations in an “executive session” without NHLBI representatives. The NHLBI also pressured the committee at times in regard to the direction its recommendations should take. A 2009 paper describing NHLBI monitoring processes stated that when the NHLBI is unhappy with recommendations from a monitoring committee, it works with the committee to develop revised recommendations that NHLBI will accept [22]. If the study sponsor is permitted to pressure its advisors regarding the recommendations they should make, the whole concept of an independent expert committee is undermined. This is particularly true when the outside experts depend on the NIH for their research funding and may therefore be reluctant to persist in advocating recommendations that the participating program staff does not like. Concern about such issues has led the US National Institute of Allergy and Infectious Diseases (NIAID) to exclude staff members with any scientific involvement in a research study from the interim monitoring process [23]. NHLBI has also recently modified its policies to permit executive sessions without the presence of any NHLBI staff, and appears to be more restrictive of NHLBI staff participation in sessions at which confidential interim data are presented [24].

Other conflicts relate to the statistician performing the interim analysis and reporting to the DMC. Traditionally, this role has been played by the primary study statistician. Difficulties can arise, however, when mid-course changes to the study are contemplated. For example, a study might be focused on improvement in heart failure, but if another study of the same or related drug reported a highly positive survival advantage with little impact on heart failure, the sponsors of the first study might want to consider changing their primary endpoint to survival. If the study statistician (who participates in the decision to change the endpoint) knows that the interim results strongly favor survival, people might question whether, had the results *not* favored survival, the statistician would have found subtle ways of discouraging the change. Such influences would compromise the interpretability of the final data.

Such situations do not occur frequently, but when they do it is important to ensure that needed changes to trial design can be made without undermining trial integrity. One solution, which has been adopted for most industry-sponsored trials and some government-sponsored trials, is to involve two statisticians in the trial, one to work with the study team on design issues and trial management, and the other to perform interim analyses and report to the DMC. This is sometimes referred to as the “independent statistician” model [25],[26] and was recommended by the FDA in its 2006 guidance document on clinical trial data monitoring committees [11]. Although this model has worked well for many trials [27],[29], a potential disadvantage is that the statistician doing the interim analysis may not be as knowledgeable about the study as the statistician involved in the design and management, potentially hampering effective communication with DMC members [28]. An alternative approach is to have the study statistician serve as the analyst for interim data, as has traditionally been done, but if changes to the design are contemplated assign a different statistician, who has not seen the interim data, to implement the change. This approach may be more appealing to many as the knowledge base of the primary study statistician remains available to the DMC, and the second statistician is not required as long as no major interim design changes are considered. As additional experience is gained with these models a consensus may emerge regarding the optimal approach, but it is clear that decision-making about mid-course changes in trial design should be done without knowledge of interim results.

## Early Termination of Trials

A long-accepted ethical principle for clinical trials is that trials should not continue if interim data provide definitive evidence of the superiority of one of the treatments. A trial whose results showed 200 deaths on one arm and five deaths on the other, for example, would be severely criticized on the grounds that a survival difference would have been evident far earlier, and many of the 200 deaths on the inferior arm should have been averted. On the other hand, in the early days of clinical trials when investigators routinely reviewed interim data, it was not uncommon for trials to stop as soon as interim findings became nominally statistically significant [30], increasing the likelihood of false positive reports and limiting the credibility of the results. Guidelines were developed during the 1970s and 1980s to permit early termination with valid claims of statistical significance [2]–[5].

A difficulty, however, is that what constitutes “definitive evidence” is inherently subjective. This issue has been debated for decades [31],[32] without resolution. Those who are more negative about early termination fear that smaller trials are less reliable and will not be widely persuasive, and therefore will not lead to changes in medical practice. A recent variation on this argument is that early termination of trials should be avoided because it leads to artificially elevated estimates of treatment effect; trials that stop early, of course, show higher estimates of effect than trials of the same regimens that do not stop early [33],[34]. Others have countered that there is nothing nefarious about the obvious fact that trials that stop early show higher treatment effects, that any upward bias is small and methods to correct the bias are readily available, and that the ethics of continuing trials of proven life-saving treatments to gain at best minor improvements in estimation of effect are questionable [35]–[38].

The implication of this debate for a DMC is that the DMC, the study sponsors, and the study investigators must all understand and be comfortable with the criteria for early termination proposed as the basis for trial monitoring, with the understanding that unanticipated issues may always arise that would lead a DMC to ignore the pre-specified criteria. For example, the efficacy boundary could be crossed, but an unanticipated safety concern could have arisen so that further study is needed to determine whether benefit outweighs the risk. Although it is well accepted that a DMC will regard stopping criteria as guidelines rather than strict rules, a DMC should be prepared to apply the agreed-upon criteria unless there are reasons to question the benefit-to-risk assessment. It has been suggested that a DMC might appropriately ignore the pre-stated stopping criteria if its members were simply skeptical that the effect was as large as that being observed [39]; this would suggest, however, that the stopping criteria were inappropriate and the DMC should have asked for more stringent criteria initially.

## Liability of DMC Members

The issue of liability coverage for DMC members has arisen as a concern only in recent years, as litigation has increasingly permeated medical research. It is difficult to cite cases; I have colleagues who have been consulted in such cases but they have all been settled with confidentiality agreements. Nevertheless, concerns on the part of DMC members have increased to the point that several NIH institutes are now providing coverage through contractors to individuals serving on their DMCs. Pharmaceutical companies are often (but not universally) willing to indemnify members of their DMCs; proposed language to be written into DMC contracts for industry studies was provided by DeMets et al. [40]. Such indemnification is probably not the optimal solution, as a DMC member's defense handled by legal staff of a pharmaceutical company could be compromised by the staff's primary responsibility to defend the company [41]. A better approach would be the availability of insurance policies that DMC members could purchase on an individual basis, with the cost of such a policy factoring into the fee a member would negotiate with a company. For an insurer to offer such policies, however, would require information about the risks it would face, and because of the limited information available the development of such policies by insurers would be challenging.

## Conclusion

DMCs have become expected components of many clinical trials, and provide an important oversight function. With increased experience, debates about best practices, as well as new issues, have emerged. New approaches are needed to ensure that DMC members do not have unacceptable conflicts of interest. Conflict of interest concerns relate also to the access to interim results. Such concerns have led to exclusion of industry representatives from involvement with interim monitoring, but the potential conflicts of noncommercial sponsors have not been adequately recognized. The “independent statistician” issue, on the other hand, seems to be resolving in favor of a model in which those making day-to-day decisions about trial design and conduct are protected from knowledge of interim results. The key to making such models successful is the presence of statisticians knowledgeable about the trial in both the trial management and the DMC reporting roles. Other issues, such as the appropriate stringency of early termination boundaries, appear less amenable to consensus, at least currently, because the characteristics of particular trials, and the philosophies of trial organizers, are too varied. The best one can hope for is that for any particular trial, the trial sponsor, investigators, DMCs, and IRBs are all in agreement about the monitoring approach taken for that trial. Finally, concerns about potential exposure of DMC members to litigation are relatively recent but need to be taken seriously by DMC members and trial sponsors. The decision of some NIH institutes to establish protection for DMC members serving on institute DMCs is a welcome development.

## Abbreviations

DMC: data monitoring committee
FDA: Food and Drug Administration
IRB: institutional review board
NHLBI: National Heart and Blood Institute
NIH: National Institutes of Health
